# Supplementary benefits of CT-guided transthoracic lung aspiration biopsy for core needle biopsy

**DOI:** 10.3389/fmicb.2022.1005241

**Published:** 2022-09-14

**Authors:** Jia-Huan He, Jia-Xing Ruan, Ying Lei, Zhi-Dan Hua, Xiang Chen, Da Huang, Cheng-Shui Chen, Xu-Ru Jin

**Affiliations:** ^1^Department of Respiratory and Critical Care Medicine, Quzhou People’s Hospital (Quzhou Hospital Affiliated to Wenzhou Medical University), Quzhou, China; ^2^Department of Respiratory and Critical Care Medicine Taizhou Central Hospital (Taizhou University Hospital), Taizhou, China; ^3^Department of Respiratory and Critical Care Medicine, The First Affiliated Hospital of Wenzhou Medical University, Wenzhou, China; ^4^Department of Radiology, The First Affiliated Hospital of Wenzhou Medical University, Wenzhou, China

**Keywords:** CT-guided lung biopsy, core needle biopsy, aspiration biopsy, pathology, microbial diagnosis

## Abstract

**Objective:**

This study aimed to investigate the diagnostic efficacy of computed tomography (CT)-guided transthoracic lung core needle biopsy combined with aspiration biopsy and the clinical value of this combined routine microbial detection.

**Materials and methods:**

We retrospectively collected the electronic medical records, CT images, pathology, and other data of 1085 patients with sequential core needle biopsy and aspiration biopsy of the same lung lesion under CT guidance in the First Affiliated Hospital of Wenzhou Medical University from January 2016 to January 2021. GenXpert MTB/RIF detection and BD BACTEC™ Mycobacterium/fungus culture were applied to identifying the microbiological results of these patients. We then compared the positive diagnostic rate, false negative rate, and diagnostic sensitivity rate of three methods including core needle biopsy alone, aspiration biopsy alone, and both core needle biopsy and aspiration biopsy.

**Results:**

The pathological results of cutting histopathology and aspiration of cell wax were examined for 1085 patients. The diagnostic rates of cutting and aspiration pathology were 90.1% (978/1085) and 86.3% (937/1085), respectively, with no significant difference (*P* > 0.05). Considering both cutting and aspiration pathologies, the diagnostic rate was significantly improved, up to 98% (1063/1085) (*P* < 0.001). A total of 803 malignant lesions were finally diagnosed (803/1085, 74.0%). The false negative rate by cutting pathology was 11.8% (95/803), which was significantly lower than that by aspiration biopsy [31.1% (250/803), *P* < 0.001]. Compared with core needle biopsy alone, the false negative rate of malignant lesions decreased to 5.6% (45/803) (*P* < 0.05). Next, the aspirates of the malignant lesions highly suspected of corresponding infection were cultured. The results showed that 16 cases (3.1%, 16/511) were infected with Mycobacterium tuberculosis complex, *Aspergillus niger*, and *Acinetobacter baumannii*, which required clinical treatment. 803 malignant tumors were excluded and 282 cases of benign lesions were diagnosed, including 232 cases of infectious lesions (82.3%, 232/282). The diagnostic rate of Mycobacterium/fungus culture for infectious lesions by aspiration biopsy (47.4%) was significantly higher than that by lung core needle biopsy (22.8%; *P* < 0.001). The diagnostic rate of aspiration biopsy combined with core needle biopsy was 56% (130/232). The parallel diagnostic rate of aspirated biopsy for GenXpert detection and Mycobacterium/fungal culture combined with core needle biopsy was 64.7% (150/232), which was significantly higher than that of lung core needle biopsy alone (*P* < 0.001). Finally, pulmonary tuberculosis was diagnosed in 90 cases (38.8%) of infectious lesions. Compared with the sensitivity of core needle biopsy to detect tuberculosis (27.8%, 25/90), the sensitivity of aspirating biopsy for GenXpert detection and Mycobacterium/fungal culture was significantly higher, at 70% (63/90) and 56.7% (51/90), respectively. Although there was no significant difference in the sensitivity of aspirated biopsy for GenXpert and Mycobacterium/fungal culture to detect pulmonary tuberculosis, the sensitivity was significantly increased to 83.3% (*P* < 0.05) when the two tests were combined. Moreover, when aspirated biopsies were combined with GenXpert detection, Mycobacterium/fungus culture, and core needle biopsy, the sensitivity was as high as 90% (81/90).

**Conclusion:**

CT-guided lung aspiration biopsy has a significant supplementary effect on core needle biopsies, which is indispensable in clinical application. Additionally, the combination of aspiration biopsy and core needle biopsy can significantly improve the diagnostic rate of benign and malignant lesions. Aspiration biopsy showed that pulmonary malignant lesions are complicated with pulmonary tuberculosis, aspergillus, and other infections. Finally, the diagnostic ability of lung puncture core needle biopsy and aspiration biopsy combined with routine microbial detection under CT positioning in the diagnosis of pulmonary infectious diseases was significantly improved.

## Introduction

Lung cancer is the leading cause of cancer-related death worldwide. The 5-year survival rate of lung cancer across all stages is only 4–17% (Nasim et al., 2019), which is mainly due to the high rates of recurrence and metastasis (He et al., 2020; Liu et al., 2021). Therefore, early diagnosis and intervention are crucial to successful treatment of lung cancer. The continuous development of computed tomography (CT) imaging technology has increased the ability to detect suspicious lung lesions, which may have otherwise been missed (Zurstrassen et al., 2020). A large number of the lung lesions found by CT are caused by infection rather than cancer, and their rapid progress can lead to systemic multiple organ failure. Due to the inability to identify the pathogen, treatment is often delayed, which is equally as life-threatening as the cancer itself. However, CT cannot accurately determine the benign and malignant lesions, which instead require the use of small biopsy or surgical pathology.

The most common methods of small lung biopsy include endobronchial ultrasound-guided biopsy, image-guided transthoracic lung biopsy, and video-assisted thoracoscopic biopsy. The combination of radiography and biopsy has developed to such an extent that image-guided transthoracic lung puncture is now considered as a safe and effective diagnostic method (Lee et al., 2019; Mallow et al., 2019; Ma et al., 2020; Chen et al., 2021), which has the advantages of high sensitivity and specificity, and low cost. CT-assisted lung puncture mainly includes cutting needle biopsy and aspiration needle biopsy. The cut specimens are subjected to histopathological analysis, while the needle aspiration specimens are commonly used for cytological evaluation. Studies have shown that the simultaneous use of both methods under CT guidance has stronger diagnostic ability than the use of one method alone. Indeed, the sensitivity and specificity are as high as 92.52% ± 3.14% and 97.98% ± 3.28%, respectively, while the puncture risk is not significantly increased (Yamagami et al., 2003; Choi et al., 2013).

Numerous studies have confirmed that core needle biopsy, also known as core biopsy, is the preferred choice for the diagnosis of malignant lung lesions. The diagnostic accuracy of pathological tissue analysis by cutting needle biopsy is higher than that by aspiration biopsy (McLean et al., 2018; Zhang et al., 2018; Sattar et al., 2019; Li et al., 2020; Tsai et al., 2020; Ye et al., 2022), but there is insufficient basis for identifying the pathogen responsible for lung infections. Lung aspiration biopsy can directly connect the aspirated tissue with the sterile culture bottle through the aspirating needle and attract the tissue through negative pressure. Compared with cutting into strip tissue, this technique can avoid the crushing and pollution of lung tissue and improve the detection rate of pathogens. However, in aspiration biopsy, the aspirate contains more bloody fluid, which affects the pathological diagnosis of aspirated cell wax and increases the false negative rate. Here, we focus on the diagnostic efficacy of lung cutting combined with aspiration biopsy, specifically, the clinical utility and potential value of combined routine microbial detection in clinical application, with the aim to provide a basis for diagnosis and decision-making.

## Materials and methods

**FIGURE 1:**
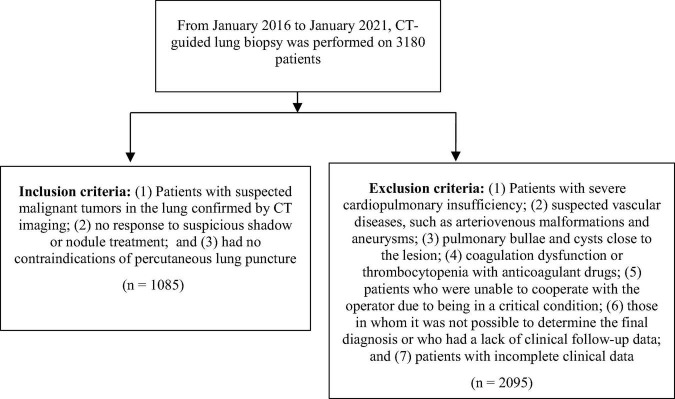
Inclusion and exclusion flowchart of the study.

We retrospectively collected the electronic medical records, CT images, pathology, and other data of 1085 patients who underwent continuous CT-guided core needle biopsy and aspiration biopsy of the same lung lesion at our institution (provincial first-class hospital) from January 2016 to January 2021. GenXpert MTB/RIF detection and BD BACTECTM Mycobacterium/fungus culture were applied to identifying the microbiological results of these patients. All included patients provided informed consent for the study. Before the biopsy, a thoracic interventional radiologist with 30 years of experience evaluated the radiological characteristics of the patients’ CT pulmonary lesions and determined the appropriate puncture point under CT positioning. The biopsy was completed by a senior pulmonary physician with extensive experience in interventional technology using a coaxial biopsy needle. Cytopathologists and Cytotechnologists were present at all of the biopsies to assess the adequacy of the samples. The inclusion criteria and exclusion criteria of this study are shown in Figure 1.

### Biopsy procedure

#### Before biopsy

For patients with lung lesions screened by CT, the necessity of lesion biopsy was first preliminarily evaluated. Next, the hospitalization was arranged, while considering the patient’s medical history in detail before biopsy. The results of blood routine examination and blood coagulation, and lung function were improved, and a puncture needle with appropriate specifications was selected. The puncture method and path were designed in advance, avoiding blood vessels, the heart, and lung bullae.

#### Biopsy procedure

The patient was placed in the supine, lateral, or prone position according to the location of the lesion. Usually, 2-mm thick spiral CT scanning was performed to determine the puncture focus, and a self-made fence-like metal surface locator was used to assist in determining the needle entry point of the chest wall skin. The skin at the puncture position was disinfected at least twice with an Iodophor cotton swab, and the diameter of the disinfection range was ≥15 cm. The operator wore sterile gloves, laid a sterile hole towel, and used a 5-ml syringe to extract 2% lidocaine for local infiltration anesthesia, being careful to avoid puncture to the blood vessel. The puncture needle was a semi-automatic combined biopsy needle (fine core biopsy needle; Nagano, Gyoda City, Saitama, Japan), with two specifications of 10 cm and 15 cm in length. The semi-automatic spring core needle biopsy gun is equipped with a cutting needle core with 18 gauge or 20 gauge and a 2-cm groove (the length of the cutting groove can be adjusted to 1 cm according to the size of the lesion). The corresponding supporting sheath tube of 17 gauge or 19 gauge was used for aspiration biopsy, and the supporting sheath tube also has a needle core to assist in pre needle insertion. First, the matching sheath and sheath needle core were inserted into the lower edge of the chest wall, before conducting CT scanning to confirm the angle and needle distance of the puncture needle before inserting the needle into the edge of the target lesion. After plain CT scanning to confirm the correct position of the needle tip, the needle core was pulled out, the length of the cutting needle core groove was preset to 1 cm or 2 cm, the needle core of the core needle biopsy gun was inserted into the sheath, and the spring plug was pressed to complete the core needle biopsy. If the operator judged the tissue to be insufficient, the core needle biopsy needle core was re-inserted for repeated operation without pulling out the sheath. The biopsy needle was generally used to puncture 2–3 times to obtain 2–3 tissues. The biopsy prints tended to be made first, before the cut specimens were placed in 10% formalin solution for histopathological evaluation. For specimens suspected to have specific infection, acid fast staining, silver hexamine staining (MSN), and Schiff periodate (PAS) staining can be used to perform further investigation. After the core needle biopsy gun was removed, the end of the tube sheath was connected with a 10-ml syringe barrel to form negative pressure suction. The aspirated tissue was placed in 10% formalin solution to prepare cell blocks for cytological evaluation. For those with clinical indications of pathogen infection and high suspicion of corresponding infection, the pulmonary physician decided whether to use part of the aspirates for microbiological examination, such as GenXpert MTB/RIF detection, BD BACTEC™ Mycobacterium/fungal culture (Becton, Dickinson and Company, USA, and BacT/Alert aerobic and anaerobic microbial culture (bioMerieux, Inc., USA). Cut tissue samples were stained with hematoxylin eosin (HE) to observe the histomorphology under the microscope for further immunohistochemical analysis, or were used for gene testing and formulating individualized treatment plans. If the cut samples were too small, immunohistochemical analysis was conducted on paraffin sections of aspirated cells to determine the subtype or source of cancer. For the lesions with unsatisfactory materials, repeat CT scanning was conducted to confirm the position of the needle tip, followed by puncture and resampling. During the operation, the patients were closely observed for signs including chest tightness, shortness of breath, palpitation, hemoptysis, severe chest pain, and other abnormalities.

#### After biopsy

After the operation, routine CT scanning was performed to observe whether there were immediate complications related to biopsy, such as pneumothorax, intrapulmonary hemorrhage, and hemoptysis. Patients rested in the examination room for ≥3 h, during which time, their vital signs were closely monitored and they underwent chest plain film to detect whether there was delayed pneumothorax within 3 h after surgery. Some cases of asymptomatic pneumothorax (more stable pneumothorax and slight blood in the sputum) can be treated conservatively, and the clinical condition can be closely observed for improvement. However, when patients present with respiratory distress and progressive pneumothorax, a thoracic drainage tube should be placed for treatment. Moreover, in cases with high levels of hemoptysis, symptomatic treatment should be given with hemostatic drugs to prevent asphyxia.

### Final diagnostic criteria

The final diagnosis was determined by a comprehensive analysis of the hospitalized patients’ electronic medical record data and clinical follow-up data. The final determination of malignant lesions was based on the following: (1) in patients who underwent surgery, the final diagnosis is surgical pathology; (2) other non-surgical biopsy pathology considers malignancy, including CT-guided lung puncture or secondary lung puncture pathology, which clearly considers malignancy, and the malignant tumor is confirmed by endobronchial ultrasound-guided transbronchial needle aspiration (EBUS-TBNA), transbronchial lung biopsy (TBLB), pleural effusion cell block, or cervical lymph node metastasis biopsy; and (3) a typical malignant growth process is observed in the clinic. Positron emission computed tomography (PET-CT) tumor imaging considers malignancy, CT image follow-up, progressive enlargement of primary lesions, and occurrence of metastases, and can be used to initiate the treatment of malignant tumors.

The final diagnosis of benign lesions was based on the following criteria, provided that the lesions had no malignant basis: (1) the biopsy lesions were confirmed to be benign by surgery and pathology; (2) the biopsy lesions were confirmed to have other benign changes determined by non-surgical biopsy pathology, such as pulmonary tuberculosis, pulmonary cryptococcosis, pulmonary aspergillosis, and hamartoma; (3) clinical imaging follow-up after discharge showed that the diameter of the lesion decreased by ≥20%, the lesion subsided, the lesion was stable for ≥24 months without special treatment (Min et al., 2009; Fontaine-Delaruelle et al., 2015; Li et al., 2020), there were no new solid components or invasive changes (e.g., short hair prick sign, lesion enlargement, pleural adhesion); (4) clear findings of microbial pathogens, such as acid-fast bacteria detected by tissue acid fast staining, Mycobacterium tuberculosis complex, non-Mycobacterium tuberculosis, pulmonary Aspergillus, or Cryptococcus cultured in lung tissue puncture or bronchoscopic alveolar lavage fluid, and genes of Mycobacterium tuberculosis were detected, which facilitated the initiation of relevant treatment; and (5) clear discharge clinical diagnosis should be considered benign. Considering benign lesions along with outpatient follow-up records, the follow-up time was generally 24 months.

In conclusion, we excluded from this analysis patients in whom it was not possible to determine the final diagnosis, or those without clinical follow-up data.

### Data collection and definition of diagnostic results

The following information was collected from the medical electronic medical record system and CT images: (1) basic data, including the patient’s age, sex, smoking history, history of extrapulmonary malignant tumor, presence of lesion cavity and type of lesion (the density under the lung window of CT image is divided into pure ground glass, partial solid, and solid), and smoking history, including never smoking (no smoking history), previous smoking (i.e., no smoking for the 3 months prior to the biopsy), and current smoking (i.e., smoking within the 3 months prior to the biopsy); (2) biopsy process data, including the patient’s body position (supine, lateral, or prone), the size of the biopsy target focus (the longest axial diameter of the cross section of the focus measured under the lung window), the lung lobe (left upper lobe, left lower lobe, right upper lobe, right lower lobe, and middle lobe or interlobular fissure) of the puncture biopsy, and the puncture depth (distance of the focus passing through the lung parenchyma along the puncture path); and (3) biopsy results, including the histopathology of the core needle biopsy, the pathology of the cell block of the aspiration biopsy, the corresponding immunohistochemical analysis, the microbial results, and complications related to biopsy [e.g., hemoptysis (excluding hemoptysis caused by primary diseases), pneumothorax, further placement of thoracic drainage tube, and other rare and serious complications].

According to the description of the pathological report, the pathological results of core needle biopsy and aspiration biopsy under CT positioning were divided into three main categories: (1) malignant, with malignant tumor cells, including heterocyst cells showing a tendency toward malignancy; (2) benign, no obvious malignant findings; and (3) insufficient specimens, such as only bloody fluid, normal lung tissue, or too few puncture objects directly indicated in the operation record.

Histopathologically positive infectious diagnosis included the following: (1) pulmonary tuberculosis, as evidenced by granulomatous inflammation with caseous necrosis and surrounding Langhans giant cells, with or without positive acid fast staining; (2) pulmonary cryptococcosis (Setianingrum et al., 2019), in which cryptococcal spores or bacteria are observed, which may manifest as granulomatous inflammation or pneumonia of multinucleated giant and epithelioid cells, with positive PAS and hexamine silver staining; (3) pulmonary aspergillosis, as shown by the Aspergillus filaments or spheroids; and (4) other pulmonary fungal (Roden and Schuetz, 2017) or bacterial infections, in which fungal filaments or bacteria can be observed under the microscope.

### Statistical analysis

When analyzing the diagnostic rate of lung puncture for infectious diseases, if the histopathology only indicates granulomatous inflammation, organized pneumonia, interstitial pneumonia, and chronic inflammation, no infectious diagnosis can be made; thus, such cases were not be included in the calculation of the positive diagnostic rate of infectious diseases in this study.

The measurement data are expressed as the mean ± standard deviation or median (range) according to whether the data were normally distributed. The count data are expressed as the rate. The sensitivity or diagnostic rate of the two methods were compared using chi-square test and McNemar’s test, and the p-value was calculated. A two-tailed *p* < 0.05 was considered to indicate statistical significance. SPSS software version 22.0 was used to conduct all statistical analyses.

## Results

### Pathological diagnostic value of core needle biopsy and aspiration biopsies

This study included 1085 cases who underwent pathological analysis of cutting histopathology and aspiration of cell block in parallel (Table 1). The median age of the patients was 63 (19–93) years, and 62.5% were male. The cases comprised 981 solid lesions (90.4%), 94 sub-solid lesions (8.7%), and 10 pure ground glass density lesions (0.9%). Moreover, there were 331 biopsy lesions with a diameter ≤20 mm, accounting for 30.5%; 369 cases with a diameter >20 mm and ≤40 mm, accounting for 34.0%; and 385 cases with a diameter >40 mm, accounting for 35.5%. We observed cavities in 5.2% (*n* = 56) of all cases. The corresponding lung lobes punctured were 22.7% (*n* = 246) in the left upper lobe, 19.9% (*n* = 216) in the left lower lobe, 23.0% (*n* = 250) in the right upper lobe, 25.1% (*n* = 272) in the right lower lobe, and 9.3% (*n* = 101) in the middle lobe or interlobular fissure. The body position distribution of patients during the operation the supine position in 397 (36.6%), the prone position in 660 (60.8%), and the lateral position in 28 (2.6%). The median puncture depth was 15 (0–79) mm. Pneumothorax occurred in 388 cases (35.8%) after puncture, of which 28 cases (2.6%) required thoracic tube drainage, while 88 cases (8.1%) had hemoptysis and recovered after conservative treatment. No rare or serious complications were found.

**TABLE 1:** General patient information related to biopsy (*n* = 1085).

Basic information	Number of cases (%)
**Age (years)**	
Median (range)	63 (19–93)
**Sex**	
Male	678 (62.5)
Female	407 (37.5)
**Smoking history**	
Never smoke	663 (61.0)
Previous smoking	227 (20.9)
Current smoking	195 (18.0)
**Puncture lung lobes**	
Left upper lobe	246 (22.7)
Right upper lobe	250 (23.0)
Middle lobe or cleft lungs	101 (9.3)
Left lower lobe	216 (19.9)
Right lower lobe	272 (25.1)
**Lesion type**	
Pure ground glass	10 (0.9)
Partial reality	94 (8.7)
Reality	981 (90.4)
**Lesion size**	
≤20 mm	331 (30.5)
20–40 mm	369 (34.0)
>40 mm	385 (35.5)
**Puncture depth** (mm)	
Median (range)	15 (0–79)
**Puncture depth**	
≤10 mm	453 (41.8)
10–30 mm	326 (30.0)
>30 mm	306 (28.2)
Cavity focus	56 (5.2)
**Puncture position**	
Supine	397 (36.6)
Prone	660 (60.8)
Lateral	28 (2.6)
**Final diagnosis**	
Malignant	803 (74.0)
Benign	282 (26.0)
**Complication**	
Pneumothorax	388 (35.8)
Thoracic tube drainage	28 (2.6)
Hemoptysis	88 (8.1)

#### Pathological diagnostic rate of core needle biopsy and aspiration biopsy

Table 2 lists the pathological classification of cut tissues and aspirated cells, which were mainly divided into malignant, benign, and insufficient. Of the analyzed lesions, 708 (65.3%) were malignant, 270 (25.0%) were benign, and 107 (9.9%) were insufficient specimens. The diagnostic rate was 90.1% (978/1085). The pathological diagnosis by aspiration biopsy identified 553 cases of malignant lesions (51.0%), 384 cases of benign lesions (35.5%), and 148 cases of insufficient specimens (13.7%), with a diagnostic rate of 86.3% (937/1085). There was no significant difference between the pathological diagnosis rate of cutting and aspiration (*P* > 0.05). Compared with the diagnostic rate of cutting or aspiration alone, when considering the pathology of both, the number of insufficient specimens decreased to 22 cases, and the diagnostic rate was significantly improved, up to 98.0% (1063/1085, *P* < 0.001).

**TABLE 2:** Pathological classification of cutting and aspiration.

Core needle biopsy tissue pathology, n (%)	Aspiration biopsy tissue pathology, n (%)			Total
	Malignant	Benign	Insufficient specimens	
Malignant	503 (46.4)	107 (9.9)	98 (9.0)	708 (65.3)
Benign	18 (1.7)	224 (20.6)	28 (2.6)	270^*b*41^ (25.0)
Insufficient specimens	32 (2.9)	53 (4.9)	22 (2.0)	107^*b*54^ (9.9)
Total	553 (51.0)	384^*b*107^ (35.5)	148^*b*143^ (13.7)	1085 (100)

^bn^ n cases were finally diagnosed as malignant, that is, n cases of malignant lesions were missed.

#### False negative rate of core needle biopsy and aspiration biopsy pathology for malignant lesions

We found no misdiagnosis of malignant tumors in the pathological dataset used in this study. The histopathology of core needle biopsy, while 41 cases (15.2%, 41/270) and 54 cases (50.5%, 54/107) were finally diagnosed as malignant. The false negative rate of malignant lesions was 11.8% (95/803). Moreover, 107 (27.9%, 107/384) and 143 (96.6%, 143/148) cases were finally diagnosed as malignant, with a false negative rate of malignant lesions of 31.1% (250/803). The false negative rate of cut tissue pathology was significantly lower than that of aspiration (*P* < 0.001); however, compared with cutting alone, when considering cutting histopathology and aspiration cell pathology, the false negative rate of malignant lesions decreased significantly (5.6%, 45/803; *P* < 0.05).

#### Clinical significance of aspiration tissue culture in malignant lesions

Among the above 803 cases of pulmonaryung malignant lesions, 511 cases (63.6%) were highly suspected of pathogen infection due to the relevant pathogen signs and clinical test results, and their aspirates were subjected to microbial culture. The results of aspirated tissue culture showed that of the 16 cases (3.1%, 16/511) with primary malignant lesions of the lung, nine cases were simultaneously infected with Mycobacterium tuberculosis complex, four cases were simultaneously infected with *Aspergillus niger*, and three cases were simultaneously infected with *Acinetobacter baumannii*; clinical intervention and targeted treatment measures were required in all cases with simultaneous bacterial infection.

### Significance of routine culture of aspirated tissue in the diagnosis of benign lesions

The above 282 cases of pathological exclusion of malignant tumors were analyzed retrospectively. The aspirates were routinely subjected to GenXpert MTB/RIF detection (Xpert) and BD BACTEC™ Myco/F lytic culture (MFC), with or without BacT/Alert aerobic and anaerobic microbial culture.

#### Classification of infectious and non-infectious benign lesions

The final diagnosis of 282 cases of benign diseases was divided into two categories. First, there were 232 cases (82.3%, 232/282) of infectious diseases, including 90 cases of pulmonary tuberculosis, three cases of atypical mycobacterial lung disease, 52 cases of pulmonary fungal infection [35 cases of pulmonary cryptococcosis, 14 cases of pulmonary aspergillosis (one of which was complicated with *Escherichia coli*), one case of pulmonary marneffei basket fungus infection, one case of cerdospora infection at the tip of the lung, and one case of pulmonary filamentous fungus infection], and 29 cases of bacterial pneumonia. Additionally, there were 58 cases of pulmonary infection with unknown pathogens; in these cases, after empirical anti-infective treatment, the CT follow-up lesions subsided significantly or the clinical symptoms were relieved, but no pathogen was found. Second, there were 50 cases of non-infectious lesions (17.7%, 50/282), including 12 cases of benign tumors (three cases of sclerosing pneumocytoma, three cases of pulmonary hamartoma, two cases of schwannoma, two cases of thymoma, one case of pleural solitary fibrous tumor, one case of inflammatory myofibroblastic tumor), one case of interstitial pneumonia, two cases of cryptogenic organic pneumonia, seven cases of pneumoconiosis, and 28 cases of other non-infectious benign lesions (lesions were stable or reduced at follow-up of ≥1 year). Among the non-infectious lesions, histopathological diagnosis included three cases of sclerosing alveolar cell tumor (100%, 3/3), two cases of pulmonary hamartoma (66.7%, 2/3), two cases of thymoma (100%, 2/2), one case of interstitial pneumonia (100%, 1/1), and seven cases of pneumoconiosis pathological changes (100%, 7/7), with one case of suspected organic pneumonia. No specific cause was found in other clinical examinations, and the final diagnosis was cryptogenic organic pneumonia (50%, 1/2). The histopathology of the remaining 34 cases (68%, 34/50) only suggested inflammatory changes and it was not possible to make a specific benign-type diagnosis. In non-infectious benign lesions, aspiration culture was negative, and no signs of infection were found in the clinical follow up.

#### Diagnostic rate of cutting pathology, aspiration biopsy for mycobacterial/fungal culture, and GenXpert for infectious lesions

The diagnostic rate of core needle biopsy for pulmonary infectious diseases was 22.8% (53/232), including 25 cases of pulmonary tuberculosis, 20 cases of pulmonary cryptococcosis, four cases of pulmonary aspergillosis, three cases of fungi, and one case of bacteria. The remaining 179 cases (77.2%, 179/232) had no specific infection diagnosis. The diagnostic rate of Mycobacterium/fungus culture in infectious lesions was 47.4% (110/232). Fifty-one cases of Mycobacterium tuberculosis complex, three cases of non-Mycobacterium tuberculosis (one case of abscess Mycobacterium and two cases of intracellular Mycobacterium), 36 cases of fungi (23 cases of Cryptococcus, 10 cases of Aspergillus, one case of filamentous fungi, one case of marneffei cyanobacteria, and one case of *Cercospora apicalis*), 20 cases of bacteria, and four cases of excluding contaminated bacteria were cultured. The diagnostic rates of mycobacterial/fungal culture of cut biopsy and aspiration biopsy were compared (Table 3), and 33 cases of infectious lesions were consistent between the two. The diagnostic rate of mycobacterial/fungal culture of aspiration biopsy was significantly better than that of cut biopsy (22.8%, McNemar’s test, *P* < 0.001). When combined with parallel diagnosis, the diagnostic rate reached 56% (130/232). The lung aspirated tissue was routinely cultured with Xpert and Mycobacterium/fungus. When either of the two results was positive, the aspirated biopsy result was considered to be positive for microbial culture. The positive number of aspirated biopsies for Xpert combined with Mycobacterium/fungus culture was 134 (57.8%, 134/232), and the positive number of both and core needle biopsies was 37 (Table 3), which was significantly higher than the diagnostic rate of core needle biopsies alone (22.8%, McNemar’s test, *P* < 0.001). The parallel diagnostic rate of the three methods was 64.7% (150/232), which was 8.7% higher than that of Mycobacterium/fungus culture combined with core needle biopsy, although without statistical significance (*P* = 0.058).

**TABLE 3:** Infectious diagnosis of core needle biopsy and aspiration biopsy (*n* = 232).

Aspiration biopsy	Result	Core needle biopsy		Total	*P*-value
		Positive	Negative		
MFC	Positive	33	77	110	*P* < 0.001*
	Negative	20	102	122	
	Total	53	179	232	
Xpert + MFC	Positive	37	97	134	*P* < 0.001*
	Negative	16	82	98	
	Total	53	179	232	

Xpert: GeneXpert MTB/RIF, MFC: BD BACTEC™ Mycobacterium/fungus culture.

*McNemar’s test.

#### Detection rate of BacT/Alert microbial culture

BacT/Alert microbial culture includes BacT/Alert fa (aerobic microorganism) and Sn (anaerobic bacteria). Fifty-seven cases (24.6%, 57/232) of pulmonary infectious diseases were sent for examination. The final diagnoses were 15 cases of pulmonary tuberculosis, one case of non-tuberculous Mycobacterium, 10 cases of fungal infection, 13 cases of bacterial infection, and 18 cases of unspecified pathogens. BacT/Alert microbial culture was positive in 10 cases (eight cases of anaerobic bacteria, one case of Legionella, and one case of Cryptococcus), and the diagnostic rate was only 17.5% (10/57). The positive diagnostic rate for core needle biopsies was 12.3% (7/57). The diagnostic rate of core needle biopsy combined with BacT/Alert microbial culture was 26.3% (15/57), which was not significantly different to that of core needle biopsy (*P* = 0.058). Comparing the results of the BacT/Alert microbial culture bottle with BD BACTEC™ Mycobacterium/fungus culture bottle (Table 4), only the detection rate of bacterial culture in the BacT/Alert microbial culture (69.2%) was higher than that in the Mycobacterium/fungus culture bottle (38.5%), but the difference was not statistically significant (*P* > 0.05).

**TABLE 4:** Results of BacT/ALERT culture and BD BACTEC™ culture (*n* = 57).

Final diagnosis	No. of cases	MFC (%)	BacT ALERT (%)
Pulmonary tuberculosis	15	7 (46.7)	0
Non-tuberculosis mycobacteria	1	1 (100)	0
Pulmonary fungal infection	10	6 (60)	1 (10)
Bacterial pneumonia	13	5 (38.5)	9 (69.2)
Pathogens not detected	18	–	–
Total	57	19 (33.3)	10 (17.5)

BacT/ALERT: BacT/ALERT microbial cultivation, MFC: BD BACTEC™ Mycobacterium/fungus culture.

#### Sensitivity of core needle biopsy, aspiration for mycobacterium culture, and Xpert in the diagnosis of pulmonary tuberculosis

According to the clinical diagnosis and follow-up results, 90 cases (38.8%, 90/232) were finally diagnosed as pulmonary tuberculosis. According to the histopathology of lung cutting, 25 cases were considered as tuberculosis, and the sensitivity was 27.8% (25/90) (Table 5). Compared with the sensitivity of cutting histopathology, the sensitivity of GenXpert MTB/RIF for aspiration biopsy was 70% (63/90), rifampicin resistance genes were detected in two cases (2.2%, 2/90), and the sensitivity of Mycobacterium/fungus culture for aspiration biopsy in the diagnosis of pulmonary tuberculosis was 56.7% (51/90), which was significantly increased (*P* < 0.05). GenXpert test and Mycobacterium/fungus culture for aspiration biopsy was both positive in 39 cases, and there was no significant difference in sensitivity (*P* > 0.05). However, compared with the single GenXpert MTB/RIF detection or Mycobacterium/fungal culture for aspiration biopsy, the combined detection identified 75 cases of Mycobacterium tuberculosis, and the sensitivity was significantly improved by 83.3% (*P* < 0.05). The sensitivity was as high as 90% (81/90).

**TABLE 5:** Diagnosis of pulmonary tuberculosis by core needle biopsy and aspiration biopsy (*n* = 90).

Core needle biopsy	Aspiration biopsy					
	Xpert		MFC		Xpert + MFC	
	Positive	Negative	Positive	Negative	Positive	Negative
Positive	16	9	15	20	19	6
Negative	47	18	36	19	56	9
Total	63	27	51	39	75	15

Xpert: GeneXpert MTB/RIF, MFC: BD BACTEC™ mycobacterium/fungus culture.

#### Sensitivity of core needle biopsy and aspiration for fungal culture in the diagnosis of pulmonary fungal infection

According to the clinical diagnosis and follow-up results, 52 cases (22.4%, 52/232) were diagnosed as pulmonary fungal infections. The results of core needle biopsy and bacterial/fungal culture for aspiration biopsy are shown in Table 6. The sensitivity of combined detection (86.5%, 45/52) was significantly higher than that of single core needle biopsy (51.9%, 27/52; *P* < 0.001).

**TABLE 6:** Diagnosis of pulmonary fungal infection by core needle biopsy and aspiration biopsy (*n* = 52).

Core needle biopsy	MFC for aspiration biopsy		Total
	Positive	Negative	
Positive	18	9	27
Negative	18	7	25
Total	36	16	52

MFC: BD BACTEC™ mycobacterium/fungus culture.

## Discussion

Transthoracic lung puncture under CT positioning is widely used in the clinic. Research has shown that the accuracy of cutting needle and aspiration needle biopsy is high, but the choice of biopsy method depends on the operator’s operation experience (VanderLaan, 2016). A recent survey of American Thoracic Radiology members showed that 85% of radiologists used cutting needles with or without suction needles for lesion biopsy (Lee et al., 2017), while there are still differences in opinion regarding whether to diagnose lung lesions by combined cutting and aspiration biopsy (Aviram et al., 2007; Schoellnast et al., 2010; Choi et al., 2013; Coley et al., 2015; Marchiano et al., 2017). This retrospective analysis showed that core needle biopsy pathology combined with aspiration biopsy cell wax pathology can significantly improve the diagnostic rate of lung puncture lesions under CT localization and reduce the false negative rate of malignant lesions. The diagnostic rate of the combination is significantly higher than that of the single core needle biopsy, indicating that the core needle biopsy and aspiration biopsy under CT positioning are complementary and reduce the insufficient rate of samples, which is critical for accurate diagnosis and effective treatment decisions relating to lung lesions. Biopsy pathology is the gold standard for diagnosing lung lesions, and, crucially, may be repeated in cases with insufficient specimens in which malignant lesions are still suspected in combination with PET-CT images. However, repeat biopsy increases CT radiation exposure as well as the inherent risks related to biopsy, such as pneumothorax, focal bleeding, fever, and chest pain, which increases the medical burden. Therefore, the combination of core needle biopsy and aspiration biopsy improves the diagnostic ability of lesions and has greater clinical benefits.

Studies have shown that core needle biopsy is the first choice for the diagnosis of malignant lung lesions. The false negative rate of cutting histopathology for malignant lesions is significantly lower than that of aspiration pathology. The analysis of pathological tissue has high accuracy in the diagnosis of malignant diseases (McLean et al., 2018; Zhang et al., 2018; Sattar et al., 2019; Li et al., 2020; Tsai et al., 2020; Yang et al., 2022; Ye et al., 2022), but it is insufficient to clarify the basis of lung infection. The diagnosis of benign diseases by cutting tissue is mainly analyzed from histopathology and corresponding special staining. When the pathological diagnosis of cutting tissue is non-specific inflammatory changes, it cannot be clearly diagnosed due to the lack of a pathogen basis. Lung aspiration biopsy can directly connect the aspirated tissue with the sterile culture bottle through the suction needle and suck it out through negative pressure. Compared with cutting tissues into strips, this method can save energy in the follow-up treatment ring, avoid crushing and pollution of lung tissue, and improve the detection rate of pathogens. However, in aspiration biopsy, due to more bloody fluid in the aspirate, a large number of red blood cells observed under the cell wax slice microscope may obscure heterotypic cells, thus increasing the false negative rate. Experienced doctors will choose to aspirate at different sites of the focus, but the judgment of operation will still be affected because the local bleeding usually shows that the focus is enlarged and the boundary is blurred on CT images. Additionally, it is easier to obtain necrotic and liquefied tissue by suction, which is not conducive to pathological analysis, affects the suction, and causes false negative. We also found that the false negative rate of malignant lesions was lower than that of cutting or aspiration alone. Chen et al. (2020) also believe that the application of two types of biopsies in the same lesion can effectively reduce false negative diagnosis, improve the diagnostic efficiency, and maximize the value of lung puncture. Clinically, most patients underwent lung biopsy to determine the nature of the lesion because they either suspected or could not rule out malignant tumor. Based on various clinical auxiliary examinations and medical history analysis, even if the small biopsy pathology does not clearly indicate malignancy, if the possibility of malignancy is high, it is still be recommended to strengthen the clinical image follow-up or consider further diagnosis and treatment. Cutting combined with aspiration biopsy pathology can maximize the diagnostic ability of malignant lesions in a single operation, which optimizes the use of medical resources, reduces false negative, achieves early diagnosis, early decision-making and treatment, and improves the survival time of patients.

Immunohistochemical staining analysis is helpful to clarify the subtypes of lung cancer (particularly poorly differentiated cancer) and understand the source of cancer. In clinical application, because cutting can obtain a relatively complete small tissue, operators prefer to cut lung tissue for immunohistochemical analysis and gene detection. However, when the focus is located in the lower lobe and close to the diaphragm, the needle tip may deviate greatly during puncture due to the large respiratory amplitude, which is not conducive to accurate positioning. For small lesions (≤20 mm) and subpleural lesions (Yu et al., 2020), the lesion tissue itself is fragile and the mucus changes, so it is difficult to cut the tissue. In such cases, the puncture passes through the lung parenchyma for a long distance, which is more likely to lead to pneumothorax and bleeding (Chen et al., 2021), affect the effectiveness of the operation, and lead to cutting failure. It is still necessary to attempt aspiration biopsy before the cutting becomes too difficult and the needle sheath is pulled out. Aspiration is the final chance to obtain suitable. Studies have shown that immunohistochemical analysis can be performed in cell wax samples (Lozano et al., 2015; Bayrak et al., 2021). In this retrospective study, we found that the immunohistochemical analysis of aspirated cell wax had a significant supplementary effect on malignant tumors.

Our results also showed that the risk of complications such as pneumothorax and hemoptysis was less when the routine suction was increased (Gupta et al., 2010b). The incidence of pneumothorax through sequential chest wall cutting and aspiration lung biopsy under CT guidance was 35.8%, which was equivalent to the 15.4–42.0% (Bae et al., 2020; Ruud et al., 2021) previously reported by interventional radiologists. Most pneumothorax can be observed conservatively, with only 2.6% requiring thoracic catheterization and drainage, which was lower than the pneumothorax catheterization rate of 4.3–7.3% reported by Heerink et al. (2017). Depending on the study population and the type of needle used, the incidence of CT-mediated transthoracic lung puncture hemoptysis ranges from 0.5 to 14.4% (Tai et al., 2016; Heerink et al., 2017). In this study, 8.1% of patients had hemoptysis, including some blood in the sputum. No serious complications, such as tumor needle metastasis, air embolism, and death, occurred. As lung suction is performed to generate negative pressure suction through the guide needle sheath after the cutting is completed, the sheath tube will be pulled out immediately after the completion, and the relevant specimens will be sent for examination without additional puncture needle placement, which will serve to reduce the operative duration. Moreover, lung aspirated tissue can be used for pathological HE staining, morphological analysis, and immunohistochemical analysis of cell blocks. Furthermore, especially in the case of insufficient cutting tissue or cutting failure, more tissue can be saved, which can also be used in subsequent molecular research (Zhao et al., 2014).

Some studies have shown that the combination of lung cutting and aspiration has no significant advantage in the diagnosis of certain inflammatory pathologies (e.g., pulmonary tuberculosis, pulmonary cryptococcosis) compared with cutting alone (Aviram et al., 2007; Schoellnast et al., 2010; Choi et al., 2013; Chen et al., 2020), and the specificity of cutting histopathology is significantly higher than that of aspiration pathology. This may be due to the histopathological evaluation of cutting needle biopsy and the cytological diagnosis of aspirates in most institutions. Histopathology can more effectively determine benign types, including tumors, under the microscope (e.g., pulmonary hamartoma, inflammatory pseudotumor, schwannoma, organized pneumonia, and granulomatous inflammation). However, in clinical practice, there remain deficiencies in the etiological diagnosis of lung cutting histopathology, with some studies showing that the sensitivity of lung cutting histopathology in the diagnosis of lung infection is 36%. Furthermore, most pathological results can only suggest chronic inflammation and cannot identify a specific infection. In lesions suspected of pulmonary infection, Kim et al. (2020) found that aspiration could detect more pathogenic microorganisms than cutting. Microbial culture is the gold standard for diagnosis, and its role in benign lesions, especially in infectious lesions, cannot be ignored. The aspirated tissue can be quickly and easily inhaled into the culture bottle through the sheath, which is simple and convenient to operate and has a low risk of pollution.

Recently, the application of aspiration in the diagnosis of infectious diseases has been increasing (Haas et al., 2017). Previous studies have found that the diagnostic rate of lung aspirate culture under CT localization for pulmonary opportunistic infection was 36.5%–80% in patients with immune impairment (Hwang et al., 2000; Carrafiello et al., 2006; Gupta et al., 2010a; Ideh et al., 2011; Hsu et al., 2012; Clement et al., 2014; Liu et al., 2022). The diagnostic rate of Mycobacterium/fungus cultures of lung aspirates was 47.4%, which was significantly higher than that of core needle biopsy. Although GenXpert MTB/RIF is only sensitive to Mycobacterium tuberculosis, the parallel diagnostic rate of GenXpert MTB/RIF detection and Mycobacterium/fungus culture for aspiration biopsy combined with lung cutting pathology is still 8.7% higher than that of Mycobacterium/fungus culture for aspiration biopsy combined with core needle biopsy. Abundant materials can be obtained by one suction for auxiliary examination. Compared with core needle biopsy alone, GenXpert MTB/RIF detection and Mycobacterium/fungus culture by increasing the suction can substantially improve the overall diagnostic rate of pulmonary infectious diseases, and reduce subsequent invasive operations and targeted antiviral therapy.

China has a high incidence of tuberculosis, with the infection rate ranking second worldwide (MacNeil et al., 2020). The hidden onset of tuberculosis has become a major burden to public health, and the situation of prevention and control is grim. Early diagnosis and treatment are crucial to controlling the progression of pulmonary tuberculosis and reducing infection. Pulmonary lesions cannot be routinely screened for Mycobacterium tuberculosis infection by CT imaging, so effective clinical detection methods are required. According to the recommendations of the World Health Organization, among all adults with suspected tuberculosis, the Xpert MTB/RIF test should be preferentially used as the initial diagnosis method. In our study, the sensitivity of the lung cutting histopathology in the diagnosis of pulmonary tuberculosis was only 27.8%, which is similar to that reported in previous studies (Montenegro et al., 2014; Jiang et al., 2016). For histopathology describing inflammation, such as granulomatous inflammation, with or without coagulative necrosis, mycobacterium culture or molecular detection must be further clarified clinically. The Xpert MTB/RIF reported in the literature shows considerable variation in the detection sensitivity of tissue samples, with a reported range of 42%–100%. This variation may be related to the heterogeneity of sampling lesions, resulting in the actual detected tissues not being the most representative samples. In this study, there was no significant difference in the sensitivity of GenXpert MTB/RIF to pulmonary tuberculosis compared to Mycobacterium/fungal culture, but the sensitivity was significantly improved when they were diagnosed in parallel (up to 83.3%). Mycobacterium tuberculosis grows slowly and requires high nutrition culture medium, with the culture taking 42 days on average. GenXpert MTB/RIF detection has high sensitivity and specificity, requires less tissue, is simple and safe to operate, and has fast detection time. Additionally, it can detect Mycobacterium tuberculosis complex DNA and rifampicin resistance within 2 h (Yu et al., 2019), which can facilitate individualized anti-tuberculosis treatment as soon as possible. Therefore, as long as conditions permit, the Xpert gene detection of lung aspirates and mycobacterium culture should be conducted in parallel to diagnose the infection early, reduce transmission (Han et al., 2021), and adopt individualized anti-tuberculosis treatment more effectively. For patients with rapid progress of tuberculosis, this combination can be employed to better control tuberculosis activity and improve prognosis.

Additionally, 57 cases underwent BacT/Alert microbial culture simultaneously, and most of the results detected anaerobic bacteria. The diagnostic rate of combined detection with histopathology did not increase significantly, which may be related to the inability of BacT/Alert FA and Sn culture bottles to culture mycobacteria and the low detection rate of fungi. The average culture time of the BacT/Alert bottle is 5 days, which cannot meet the growth time of most fungi, whereas the culture time of the BD BACTEC™ Mycobacterium/fungus bottle is 42 days. The detection rate of BacT/Alert microbial culture bacteria is higher than that of the BD BACTEC™ Mycobacterium/fungus culture bottle, which is related to the detection of obligate anaerobic bacteria, while the BD BACTEC™ Mycobacterium/fungus culture bottle cannot detect obligate anaerobic bacteria. Despite no statistical difference between the two, this may have relevance for selecting clinical antibiotics. The results of this study suggest that GenXpert MTB/RIF detection, BD BACTEC™ Mycobacterium/fungus culture, and BacT/Alert specific anaerobic bacteria culture should be conducted routinely to maximize the detection rate of pathogens.

In this study, 20.6% of pulmonary infections did not identify the pathogen in other auxiliary examinations, such as blood culture, related serum antibody detection, and bronchoscopic interventional diagnosis and treatment, and the CT follow-up lesions subsided significantly or the clinical symptoms were relieved after empirical anti-infection treatment. The early use of antibiotics may make it difficult to detect sensitive bacteria in the follow-up examination. Additionally, many microorganisms are difficult to cultivate and require specific culture medium and strict culture conditions, such as Brucella, Chlamydia trachomatis, flagellin spirochete, and Neisseria gonorrhoeae (Glaser and Montone, 2020). Aspiration culture should be performed as soon as clinically feasible. Additionally, molecular technology, such as high-throughput sequencing, is not restricted by culture conditions and can directly detect nucleic acids, which is worthy of popularization.

In 50 cases of benign non-infectious lesions, the histopathological diagnosis was consistent with the imaging and clinical analysis. Indeed, the clinical CT follow-up was sufficient to exclude malignancy and consider the lesion to be benign. The combination of cutting histopathology and aspiration microbial detection is more conducive to the diagnosis of benign lesions, with the exception of infection (Hwang et al., 2000). In this study, in 3.1% of malignant lesions, infectious bacteria was detected simultaneously, including *Mycobacterium tuberculosis*, *Aspergillus*, and *Acinetobacter baumannii*. For patients with cancer, timely effective anti-infection programs will be crucial to control the spread and progress of infection.

Aspiration has a unique complementary value in the diagnosis of benign and malignant diseases. Aspiration is an easy means to process microbial samples and plays a decisive role in the diagnosis of infectious pathogens. However, this study has the following limitations: (1) it is a retrospective study, only evaluated the lung puncture data of one hospital, was limited to the operation of a single biopsy needle, and did not analyze the situation of other institutions; (2) we could not directly compare the differences in immunohistochemistry in histopathology and cell pathology because immunohistochemical analysis is the first choice of histopathological specimens, and cell pathology is often used as auxiliary research, especially when histopathology is insufficient; and (3) due to the lack of corresponding standards, the local bleeding of lesions observed on CT images was not graded and evaluated.

## Data availability statement

The raw data supporting the conclusions of this article will be made available by the authors, without undue reservation.

## Ethics statement

Ethical review and approval was not required for the study on human participants in accordance with the local legislation and institutional requirements. Written informed consent for participation was not required for this study in accordance with the national legislation and the institutional requirements.

## Author contributions

X-RJ conceived and designed this study. J-HH and J-XR performed the analyses and wrote the manuscript. YL, Z-DH, XC, DH, and C-SC performed the literature review and assisted with data collection. All authors have read and approved the final manuscript.
